# Wastewater treatment plants, an “escape gate” for ESCAPE pathogens

**DOI:** 10.3389/fmicb.2023.1193907

**Published:** 2023-05-24

**Authors:** Luminita Gabriela Marutescu, Marcela Popa, Irina Gheorghe-Barbu, Ilda Czobor Barbu, Daloha Rodríguez-Molina, Fanny Berglund, Hetty Blaak, Carl-Fredrik Flach, Merel Aurora Kemper, Beate Spießberger, Laura Wengenroth, D. G. Joakim Larsson, Dennis Nowak, Katja Radon, Ana Maria de Roda Husman, Andreas Wieser, Heike Schmitt, Gratiela Pircalabioru Gradisteanu, Corneliu Ovidiu Vrancianu, Mariana Carmen Chifiriuc

**Affiliations:** ^1^Department of Microbiology and Immunology, Faculty of Biology, Research Institute of the University of Bucharest, University of Bucharest, Bucharest, Romania; ^2^Earth, Environmental and Life Sciences Section, Research Institute of the University of Bucharest, University of Bucharest, Bucharest, Romania; ^3^Institute and Clinic for Occupational, Social and Environmental Medicine, University Hospital, LMU Munich, Munich, Germany; ^4^Institute for Medical Information Processing, Biometry, and Epidemiology – IBE, LMU Munich, Munich, Germany; ^5^Pettenkofer School of Public Health, Munich, Germany; ^6^Department of Infectious Diseases, Institute of Biomedicine, University of Gothenburg, Gothenburg, Sweden; ^7^Centre for Antibiotic Resistance Research in Gothenburg (CARe), University of Gothenburg, Gothenburg, Sweden; ^8^Centre for Infectious Disease Control, National Institute for Public Health and the Environment, Bilthoven, Netherlands; ^9^German Centre for Infection Research (DZIF), Partner Site Munich, Munich, Germany; ^10^Max von Pettenkofer Institute, Faculty of Medicine, LMU Munich, Munich, Germany; ^11^Department of Infectious Diseases and Tropical Medicine, LMU University Hospital Munich, Munich, Germany; ^12^Comprehensive Pneumology Center Munich (CPC-M), German Center for Lung Research (DZL), Munich, Germany; ^13^Romanian Academy of Sciences, Bucharest, Romania; ^14^The Romanian Academy, Bucharest, Romania

**Keywords:** ESCAPE pathogens, antibiotic resistance, wastewater treatment plants, antibiotic resistance genes, ESCAPE species dissemination, high-risk clones, multidrug resistance

## Abstract

Antibiotics are an essential tool of modern medicine, contributing to significantly decreasing mortality and morbidity rates from infectious diseases. However, persistent misuse of these drugs has accelerated the evolution of antibiotic resistance, negatively impacting clinical practice. The environment contributes to both the evolution and transmission of resistance. From all anthropically polluted aquatic environments, wastewater treatment plants (WWTPs) are probably the main reservoirs of resistant pathogens. They should be regarded as critical control points for preventing or reducing the release of antibiotics, antibiotic-resistant bacteria (ARB), and antibiotic-resistance genes (ARGs) into the natural environment. This review focuses on the fate of the pathogens *Enterococcus faecium*, *Staphylococcus aureus*, *Clostridium difficile*, *Acinetobacter baumannii*, *Pseudomonas aeruginosa*, and *Enterobacteriaceae spp.* (ESCAPE) in WWTPs. All ESCAPE pathogen species, including high-risk clones and resistance determinants to last-resort antibiotics such as carbapenems, colistin, and multi-drug resistance platforms, were detected in wastewater. The whole genome sequencing studies demonstrate the clonal relationships and dissemination of Gram-negative ESCAPE species into the wastewater via hospital effluents and the enrichment of virulence and resistance determinants of *S. aureus* and enterococci in WWTPs. Therefore, the efficiency of different wastewater treatment processes regarding the removal of clinically relevant ARB species and ARGs, as well as the influence of water quality factors on their performance, should be explored and monitored, along with the development of more effective treatments and appropriate indicators (ESCAPE bacteria and/or ARGs). This knowledge will allow the development of quality standards for point sources and effluents to consolidate the WWTP barrier role against the environmental and public health AR threats.

## 1. Introduction

The discovery of antibiotics was one of the most significant medical achievements of the 20th century, saving many lives and contributing to the control of numerous infectious diseases. The majority of the antibiotics that are used today were discovered in the period referred to as the golden age, i.e., 1940–1960, when at least 20 clinically relevant classes were developed (Katz and Baltz, 2016; O’Neill, 2016; Hutchings et al., 2019). However, the existing classes of antibiotics act selectively against only a few microbial cell targets: cell wall, plasma membrane, synthesis of proteins and metabolites, or DNA transcription and replication, allowing the bacterial species to develop resistance mechanisms during the millions of years of coevolution. Although many international authorities advocate for incentives to encourage the development of new antibiotics,^1^ (The Joint Programming Initiative on Antimicrobial Resistance, 2018) the interest of pharmaceutical companies in developing novel antibiotics has dramatically declined. Thus, in the last decades, only two new antibiotic classes (lipopeptides and oxazolidinones) providing coverage only against Gram-positive bacteria have been developed and approved, the other new molecules being analogs of the existing classes, drugs repurposed for the treatment of infectious diseases, non-antibiotics or antibiotic adjuvants (Xie et al., 2022).

The natural ability of bacteria to develop resistance has been accelerated by the selective pressure exerted by the improper use of antibiotics in human therapy, animal husbandry, and agriculture. Between 2000 and 2010, global antibiotic use increased by 36%, reaching 45% in the case of carbapenems, and the amount of all antibiotics used in animal food was estimated to be 200,235 tons in 2030 (Dhingra et al., 2020; Boyd et al., 2021). The acute limitation of currently available therapeutic options leads to increased morbidity and mortality rates, longer treatment duration, and higher hospitalization costs, questioning the efficacy of modern medical practices, which will become very risky because of common infections. Worldwide, it is estimated that 4.95 million deaths were associated with and 1.2 million attributable to bacterial AMR in 2019, mainly from lower respiratory infections and involving six leading pathogens (*E. coli*, *S. aureus*, *K. pneumoniae*, *Streptococcus pneumoniae*, *A. baumannii*, and *P. aeruginosa*) (Murray et al., 2022).

Clinical surveillance programs indicate that the prevalence of human pathogens exhibiting multidrug (MDR), extended drug (XDR), or pan-drug (PDR) resistance is rising to dangerously high levels in all parts of the world (Cheesman et al., 2017). The most threatening resistant pathogens are known under different acronyms, such as ESKAPE (E) [*Enterococcus faecium*, *Staphylococcus aureus*, *Klebsiella pneumoniae*, *Acinetobacter baumannii*, *Pseudomonas aeruginosa*, *Enterobacter* sp. (*Escherichia coli*), ESCAPE or AmpC-producing SPICE (*Serratia*, *Providencia*, indole-positive *Proteus*, *Morganella*, *Providencia* species/*Acinetobacter*, *Citrobacter*, *Enterobacter* species) or are included in different “black” lists (Rice, 2008; Moy and Sharma, 2017)]. In addition, the WHO published the critical/high/medium priority pathogens list for R&D of new antibiotics, including carbapenem-resistant *A. baumannii* and *P. aeruginosa*, carbapenem-resistant and extended-spectrum beta-lactamase (ESBL)-producing *Enterobacteriaceae*, clarithromycin-resistant *Helicobacter pylori*, fluoroquinolone-resistant *Campylobacter* spp., *Salmonella* and *Shigella*, cephalosporin-resistant, fluoroquinolone-resistant *Neisseria gonorrhoeae*, ampicillin-resistant *Haemophilus influenzae*, vancomycin-resistant *E. faecium*, methicillin-resistant, vancomycin-intermediate, and resistant *S. aureus*, and penicillin-non-susceptible *Streptococcus pneumoniae* (WHO, 2017; Asokan et al., 2019). Furthermore, with the increasing use of whole-genome sequencing to analyze antibiotic-resistant pathogens, it has become clear that many of the significant resistance problems are associated with a few successful bacterial clones within a species, with worldwide dissemination in hospitals and, possibly, in the community and the natural environment (Oliver et al., 2015; Andersson and Hughes, 2017; MacLean and San Millan, 2019).

**FIGURE 1:**
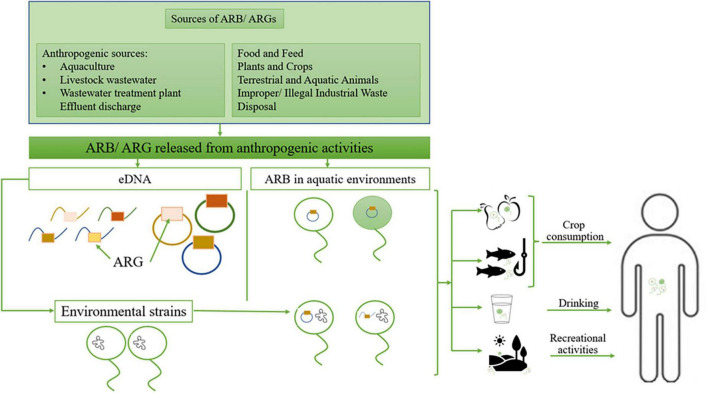
The environment role as a source of resistant pathogens; ARB, antibiotic-resistant bacteria; ARG, antibiotic resistance genes; eDNA, environmental DNA. Adapted from Denissen et al. (2022).

Numerous scientific publications reported the presence in the natural environment of clinically relevant antibiotic-resistant bacteria (ARB) and antibiotic-resistance genes (ARG) (Nnadozie and Odume, 2019; Pazda et al., 2019). Thus, the environment can serve as a source/reservoir of already resistant pathogens or may acquire ARGs from other human / animal-associated bacteria or the environmental resistome. Further, these ARGs could be transferred to the clinic and vice versa (Figure 1).

The potential gene exchange between environmental and clinical resistomes through horizontal gene transfer (HGT) involves mobile genetic elements (MGE) such as plasmids, integrative and conjugative elements (ICE), transposons, and integrons (Singer et al., 2016; Bengtsson-Palme et al., 2018; Larsson and Flach, 2022). The HGT occurs mainly via three mechanisms: conjugation (transfer of DNA between bacteria by direct cell-to-cell contact, considered the main AMR dissemination strategy), natural transformation (uptake of the free DNA from the environment), and transduction (transfer of DNA between bacteria via a bacteriophage) (von Wintersdorff et al., 2016; Chen et al., 2018). The MGE, such as integrons, are frequently associated with multi-drug resistance (MDR), increasing the risk of co-selection and persistence of multiple resistance determinants under the selective pressure of antimicrobial agents (Deng et al., 2015).

Many environmental ARGs have been shown to possess a high sequence similarity to those in human / domesticated animal fecal microbes (Nesme et al., 2014) and human/animal pathogens (Forsberg et al., 2012). For example, the quinolone resistance gene *qnr*A was first detected in clinical isolates of *K. pneumoniae* in the USA and then found in the aquatic Gram-negative bacteria *Shewanella algae* and in *Aeromonas* spp. isolated from the Seine River (France) (Cattoir et al., 2008; Perry and Wright, 2013). Most of the ARGs with known origins were likely mobilized from their native context by MGEs, such as IS/ISCR elements (Ebmeyer et al., 2021). However, their recent origin is still unknown for the great majority of the ARGs, probably because the original environmental bacterial hosts have not yet been sequenced (Larsson and Flach, 2022).

The “One Health” approach published in 2017 reinforced the importance of addressing the AMR issue outside the clinical sector by encompassing the environmental contribution to the emergence, accumulation, and spread of AMR (Fletcher, 2015; Baker et al., 2018; MacLean and San Millan, 2019). In order to map and understand the transmission routes of ARGs among clinical and environmental bacteria, research on the distribution patterns of ARGs and their temporal dynamics in different microbiomes and studies tracking the acquisition of ARGs in complex communities are required (Andersson and Hughes, 2017; MacLean and San Millan, 2019; Surleac et al., 2020).

There are numerous reports on the detection of wastewater effluents and impacted rivers of a wide variety of clinically significant ARB (Zhang et al., 2009; Giebułtowicz et al., 2018; Limayem et al., 2019).

This review aims to present an update regarding the contribution of WWTPs to the antibiotic resistance reservoir, focusing on ESCAPE pathogens and associated ARGs. We performed a literature search in Pubmed, Scopus, and Google Scholar databases using the combinations of the following keywords: ESCAPE pathogens (searched both as abbreviation and individually for each pathogen) + antibiotic resistance / wastewater treatment plants / antibiotic resistance genes / wastewater / high-risk clones / multidrug resistance. Consequently, a title and abstract screening was performed, and the papers published in the last 10 years, as well as some significant studies published previously, have been considered. The articles duplicated, irrelevant, and without full text were excluded. The remaining studies have been grouped into six categories, corresponding to each ESCAPE pathogen species and one last category, referring to the abundance of ARGs in WWTPs environments and the efficiency of different wastewater treatments in decreasing or removing ARGs.

## 2. WWTPs—An escape gate for ESCAPE pathogens and associated ARGs

Wastewater treatment plants receive a continuous discharge of human-associated ARB, ARG, antibiotics, disinfectants, and metals, which are co-locating inside the WWTP, together with the commensal microbiota, generating the perfect premises for antibiotic resistance emergence, accumulation, and dissemination (Bougnom and Piddock, 2017; Karkman et al., 2019; Kraemer et al., 2019; Paulshus et al., 2019; Pärnänen et al., 2019; Reichert et al., 2019).

Although WWTPs reduce the number of total bacteria released into the environment, the current wastewater treatment technologies do not altogether remove and/or biodegrade ARB, ARG, and antibiotics (Jäger et al., 2018; Müller et al., 2018). The sewage’ inner surfaces favor the colonization with wastewater-derived microorganisms that develop biofilms, which are continuously exposed to antibiotic residues and ARB from wastewater (Auguet et al., 2017). An analysis of the biofilm-embedded bacteria from influent and effluent sewage showed a lower susceptibility to antibiotics than bacteria from wastewater (Lépesová et al., 2018). Moreover, biofilms could facilitate the HGT, as shown by an *in vitro* study, demonstrating an increased transfer rate of the plasmidial CTX-M-15 gene in a *K. pneumoniae* biofilm compared to planktonic growth conditions (Hennequin et al., 2012). The transfer of conjugative transposons carrying ARG, such as Tn916, might also be responsible for acquiring resistance mechanisms in biofilm bacteria (Hannan et al., 2010). Microfluidic systems combined with laser confocal microscopy, fluorescent labeling of bacteria, and flow cytometry have been developed to investigate the HGT of ARGs in activated sludge biofilm (Qiu et al., 2018). WWTP discharges might also impact the river-streambed biofilm communities that often prevail in natural environments compared to free, planktonic bacteria (Proia et al., 2016). The released WWTPs effluents could impact the river biofilms through the fixation of pharmaceuticals and the persistence of the discharged microorganisms in the streambed biofilms (Aubertheau et al., 2017; Subirats et al., 2017). It has been shown that some ESCAPE pathogens (enterococci, *A. baumannii*, and *P. aeruginosa*), as well as associated ARGs (*erm*B, *bla*TEM, *tet*M, and *qnr*S), are associated with the particulate fraction of the WWTP effluent and are more probably remaining in the riverbed of the receiving water due to sedimentation (Brown et al., 2019). However, little information is available regarding the ARG distribution in the river streambed and how wastewater-associated microorganisms, including potential pathogens, contribute to maintaining the streambed resistome.

Wastewater systems, especially those receiving hospital or livestock wastewater, were demonstrated to be significant sources of significant epidemic pathogens belonging to the so-called high-risk clones: *E. coli* sequence type (ST) 131 (Finn et al., 2020), *E. faecium* HiRECC (Sadowy and Luczkiewicz, 2014), *Acinetobacter* IC2 carrying OXA-23, and IC1 carrying OXA-72 (Goic-Barisic et al., 2017; Higgins et al., 2018), *K. pneumoniae* ST11, and ST258 (Surleac et al., 2020), and *P. aeruginosa* ST235, ST111, and ST175. Furthermore, increased correlations of ESCAPE pathogens with clinically relevant ARGs (e.g., *bla*NDM-1, *van*A) were found in WWTP effluents influenced by hospital wastewaters. Furthermore, isolates carrying the *mcr-1* gene, providing resistance to the last resort antibiotic colistin, have been reported in WWTP sewage, probably originating from food-producing animals (Ovejero et al., 2017). Additionally, the selective pressure within the wastewater treatment plants (WWTP)/sewer systems, an ecosystem with consistent exposure to antibiotics and other chemical pollutants such as biocides, heavy metals, etc., could contribute to the emergence of new antibiotic-resistant variants, with possible epidemic risk (Flach et al., 2018; Danner et al., 2019; Kraupner et al., 2021). In a study aimed to assess the presence of ESKAPE strains in process water from delivery and unclean areas as well as wastewater from the in-house WWTPs of German poultry slaughterhouses, at least one of the target species was detected in 87.5% of the wastewater samples and 86% of the process water samples, with the following decreasing order of their prevalence: *E. coli* > *A. calcoaceticus-A. baumannii* (ACB) complex > *S. aureus* > *K. pneumoniae* > *Enterobacter* spp., *Enterococcus* spp., and *P. aeruginosa* (Savin et al., 2020).

In the next subchapters we will present an update of the current knowledge on the ESCAPE pathogens to WWTP relationships.

### 2.1. *Enterococcus faecium*

Enterococci are ubiquitous in nature, found in soil, plants, surface water, wastewater, and food, as well as in the gastrointestinal tract of animals and humans (Sanderson et al., 2020). *E. faecium* and *E. faecalis* are the predominant species in wastewater, likely due to the continuous input of fecal waste into these systems. Comparative genomics indicated a lower diversity of *E. faecium* isolates in the wastewaters than *E. faecalis*, suggesting that *E. faecium* isolates may be more adapted specifically to clinical environments from which they are released in the natural environment (Sanderson et al., 2020).

Łuczkiewicz et al. (2010) reported a significant increase in resistance prevalence, after water treatment, for both *E. faecalis* and *E. faecium*, to fluoroquinolones (ciprofloxacin, levofloxacin, and moxifloxacin), tetracycline, and erythromycin. The *E. faecium* and *E. faecalis* isolates were expressing high-level resistance to aminoglycosides (up to 4.5% for gentamicin) and glycopeptides (for teicoplanin up to 2.7% and for vancomycin up to 6.8%) (Łuczkiewicz et al., 2010). *E. faecium*, with high prevalence of resistance to ciprofloxacin, was reported to be positively selected during WWTP treatment (Ferreira da Silva et al., 2006).

*Enterococcus faecium* belonging to a high-risk clonal group named clonal complex-17 (CC17), is associated with clinical infections and hospital outbreaks worldwide, exhibiting increased levels of ciprofloxacin resistance and carrying the *esp* gene associated with adhesion to cells and pathogenicity (van Schaik et al., 2010). This nosocomial clone may survive and pass through the wastewater system reaching the environmental reservoirs (Leclercq et al., 2013; Sadowy and Luczkiewicz, 2014; Sanderson et al., 2020). Leclercq et al. (2013) reported the identification of a large number of *E. faecium* strains belonging to the CC17 complex resistant to ciprofloxacin and carrying acquired macrolide resistance genes in hospital and retirement home effluents. In Gauteng, South Africa, isolates of *E. faecium* CC17 clonal complexes were recovered only from hospital sewage but were not detected further in WWTP and surface waters (Hamiwe et al., 2019).

Enterococci were demonstrated to survive in beach sediments, thus their release from untreated wastewater into the sea could pose a potential threat to the health of recreational users (Anderson et al., 2015); thus, the release of treated and into the sea. In marine outfalls directly impacted by treated wastewater, a lower number of enterococci by four orders of magnitude (<100 CFU/100 mL) was reported in comparison to corresponding treated wastewater, which is below the value described by the New Bathing Water Directive 2006/7/EC for coastal water of “excellent quality.” However, in WWTP effluents and marine outfalls, the isolates belonging to the significant nosocomial HiRECC (formerly CC17) constituted 24.6% of all E. faecium. Furthermore, these isolates were resistant to ciprofloxacin and ampicillin and showed an MDR phenotype in the vast majority (Sadowy and Luczkiewicz, 2014).

It has been shown that planktonic *Enterococcus* spp. isolated from influent wastewater exhibited low resistance to ampicillin and ciprofloxacin, while those recovered from the influent biofilm were resistant to vancomycin, and most were MDR (Lépesová et al., 2018). In the Netherlands, ampicillin-resistant *E. faecium* strains with intermediate vancomycin resistance have been isolated from WWTP-treated effluent but not from the receiving surface water (Taučer-Kapteijn et al., 2016).

Multidrug vancomycin-resistant enterococci (VRE) were detected in unchlorinated effluent samples, suggesting an exposure risk for WWTP workers (Rosenberg Goldstein et al., 2014). The majority of *E. faecium* isolates (86%) from the WWTP effluent in the Czech Republic harbored the *van*A gene and belonged to ST17, ST18, and ST78, while those isolated from the WWTP effluent and downstream in Poland (57%) harbored *van*C1 (27.6%) (Oravcova et al., 2017; Gotkowska-Płachta, 2021).

Vancomycin-resistant enterococci have been used as indicators of antimicrobial resistance in two WWTPs from the same municipality, one with a biologically aerated filter (BAF) and the other with conventional activated sludge (CAS). The BAF system assured a better removal rate of total enterococci, VRE, and levofloxacin resistance, while CAS was selected for nitrofurantoin resistance and reduced quinupristin/dalfopristin and streptomycin-resistant enterococci (Sanderson et al., 2019).

The results of the current studies indicate an incomplete removal of resistant enterococci, including VRE strains belonging to clinically significant sequence types, during wastewater treatment.

### 2.2. *Staphylococcus aureus*

Drug-susceptible and drug-resistant *S. aureus* have been identified in the US’s influent and effluent samples collected from four WWTPs (Goldstein et al., 2012). From the very few studies documenting the release of methicillin-resistant *S. aureus* (MRSA) through wastewater systems into the environment (Börjesson et al., 2009; Boopathy, 2017; Kozajda and Jeżak, 2020), it appears that hospital wastewaters add to the load of MDR *S. aureus* entering WWTP. In a study performed in Poland, among 149 *S. aureus* isolates (2 from the air and 147 from wastewater), only two were MRSA, and over 60% were resistant to penicillin. In contrast, ∼20% showed MDR phenotypes (Kozajda and Jeżak, 2020). Thompson et al. (2013) reported detecting one MRSA isolate resistant to nine antibiotics in hospital effluent. MRSA ST398 carrying the *mec*A gene and exhibiting an MDR phenotype (resistance to clindamycin, ciprofloxacin, tetracycline, and aminoglycosides) was detected in an effluent water sample in Spain (Gómez et al., 2016). A comparative study of the MRSA prevalence and the genotypic and phenotypic characteristics of MRSA isolates from a municipal (M) and a swine slaughterhouse (S) WWTP revealed a different profile of the *SCCmec* types in the two types of WWTP and higher abundance of *mec*A gene in the S-WWTP (Wan and Chou, 2015).

In one study, although wastewater treatment reduced the number of MRSA isolates and diversity, the remaining strains exhibited a higher resistance level and virulence (the presence of the PVL gene) (Börjesson et al., 2009). Both hospital and community-related clonal complexes have been detected in fully treated WWTP effluents. The genetic analysis of the *S. aureus* isolates obtained after treatment showed diversity in the *spa* type and carriage of MGEs, suggesting that treatment could facilitate persistence, evolution, and genetic shifts (Amirsoleimani et al., 2019). A recent study evaluated the occurrence and environmental health risk of *S. aureus* and MRSA from hospital effluent to sewage treatment plant (STP) and finally to river water at the basin level; a high abundance of over 90% has been detected in the sewage treatment plant, and the contribution of the pollution load derived from the target STP effluent to river water ranged from 2 to 25% (Azuma et al., 2022).

The few available studies report the presence of multidrug-resistant *S. aureus* and MRSA belonging to different clonal complexes in the WWTP effluents and surface water, suggesting the importance of reducing or inactivating *S. aureus* and MRSA before the effluent is discharged into rivers.

### 2.3. *Clostridioides difficile*

More than 25% of the hospital- and community-associated *Clostridioides difficile* infections originate in community sources such as asymptomatic carriers, animals, food, and WWTP (Hensgens et al., 2012; Warriner et al., 2017). In addition, the resistance genes to metronidazole and vancomycin antibiotics used for treating *C. difficile* infections, i.e., the *nim* and *van*A genes, were detected in wastewater decades ago (Trinh and Reysset, 1996).

*Clostridium difficile* is a chlorine-resistant, spore-forming bacterium that can survive in contaminated aquatic environments such as wastewater (Baghani et al., 2020). *C. difficile* was detected in 11.8% of untreated human wastewater samples in a study performed in Texas (Norman et al., 2011) and in 96% of anaerobically digested sludge samples, 92% of raw sludge, and 73% of dewatered biosolids and effluent discharges from two Southern Ontario WWTPs (Xu et al., 2014). The analysis of a conventional activated sludge treatment plant and a waste stabilization pond system in Iran revealed the presence of *C. difficile* in 13.6% of digested sludge samples and 5% of the waste stabilization ponds samples, all strains being toxigenic (positive for the tcdB gene) (Nikaeen et al., 2015). Resistant hypervirulent *C. difficile* (ribotype 078 or toxinotype V) strains have been detected in the WWTPs influents and effluents in China, and the raw sewage, digested sludge, and biosolids from Southern Ontario WWTPs (Xu et al., 2014). The *C. difficile* isolates from raw sewage influents and treated effluents of WWTPs from southern Switzerland exhibited a large diversity, belonging to 13 different known ribotypes (009, 010, 014, 015, 039, 052, 053, 066, 070, 078, 101, 106, and 117), to which non-typeable strains were added. Eight ribotypes (010, 014, 015, 039, 066, 078, 101, and 106) were also detected in hospitalized symptomatic patients in the respective region. The most frequently isolated ribotype (40%) was the hypervirulent ribotype 078, present in more than 50% of the sampled WWTPs (6/9). In contrast, the toxigenic emerging ribotype 066, associated with hospital infection, was isolated from the effluent of one plant. Most isolated strains (85%) were toxigenic, with 49% harboring the profile A + B + CDT + and 51% the profile A + B + CDT–(Romano et al., 2012). In another study performed in Slovenia, *C. difficile* was detected in all analyzed WWTP samples, the recovered isolates belonging to 32 different ribotypes, of which 014/020 and 010 were the most prevalent (Steyer et al., 2015). The analysis of 18 WWTPs from across the East of England, half of which were located downstream of hospitals, has revealed the presence of *C. difficile* in the effluent of all WWTPs, belonging to 38 STs, out of which 13 were common to clinical isolates analyzed in the same temporo-spatial sequence (Moradigaravand et al., 2018). In New Zealand, toxigenic *C. difficile* has also been detected with high frequency in wastewater (10 out of 13 toxigenic isolates), belonging to eight PCR-ribotypes (RTs), including two novel RTs (878 and 879). However, all *C. difficile* isolates were susceptible to the first-line human antimicrobials used to treat *C. difficile* infection (Rivas et al., 2020).

These studies confirm the extensive escape of toxigenic and resistant *C. difficile* from WWTPs into surface waters, raising the need to monitor these bacteria in treated wastewater.

### 2.4. *Acinetobacter baumannii*

*Acinetobacter baumannii*, a leading cause of nosocomial infections, hospital outbreaks, and sporadic acute community-acquired infections with severe evolution in critically ill patients, is known for its ability to develop resistance to multiple antibiotics (Yakkala et al., 2019). MDR *A. baumannii* was reported in untreated hospital wastewater in India, Brazil, China, and Croatia (Ferreira et al., 2011; Zhang et al., 2013; Seruga Music et al., 2017; Marathe et al., 2019). In a study performed in China, *Acinetobacter* spp. isolates from urban WWTPs effluents were found to express high resistance to rifampin (72.4%), chloramphenicol (69%), and amoxicillin plus clavulanic acid (37.9%), and 84.5% of the tested isolates were MDR (Zhang et al., 2009).

Goic-Barisic et al. (2017) reported the release of hospital wastewaters containing clinical carbapenem-resistant *A. baumannii* to the Adriatic Sea without any pre-treatment, as this is not legally required in Croatia. Carbapenem-resistant isolates belonging to international clonal lineage IC2 carrying OXA-23, IC1 carrying OXA-72, and even pan-drug resistant isolates were detected in the WWTP effluent (Goic-Barisic et al., 2017; Higgins et al., 2018). In addition, carbapenem-resistant *A. baumannii* isolates were detected in the urban sewage receiving hospital wastewater and the river (Seruga Music et al., 2017; Higgins et al., 2018). The prevalence of carbapenem-resistant *A. baumannii* and ARGs in untreated and treated wastewater has been analyzed for three consecutive seasons; during 2019, in Poland, the highest prevalence of target bacteria was recorded in the wastewater collected in June and September, as compared to February. The ISAba1/blaOXA-51 complex associated with carbapenem resistance was identified in 13 isolates. The number of resistant *Acinetobacter* isolates increased in river water samples collected downstream compared to upstream from the WWTP (Hubeny et al., 2022). A study performed on *A. baumannii* isolated in 2018 and 2019 from hospital settings, hospital collecting sewage tanks, and the receiving WWTPs located in the central geographical regions of Romania has shown that the strains isolated from hospital effluents belonged to epidemic clones, such as ST2 and exhibited high MDR rates. The WGS analysis revealed the relatedness between clinical and hospital wastewater strains and the possible dissemination of clinical *A. baumannii* belonging to ST2 in the wastewater (Gheorghe-Barbu et al., 2022).

The current evidence shows that WWTP might be a source of dissemination in the environment of *Acinetobacter* strains carrying clinically significant ARGs, including carbapenem-resistance genes.

### 2.5. *Pseudomonas aeruginosa*

*Pseudomonas aeruginosa* is an environmental bacterium that can cause human infections, particularly in patients with compromised host defense mechanisms. It is associated with urinary, gastrointestinal, soft tissue, bone, joint, and surgical site infections (European Centre for Disease Prevention and Control, 2022). The MDR/XDR *P. aeruginosa* high-risk clones such as ST235, ST111, and ST175, associated with chronic and hospital-acquired infections with significant morbidity and mortality, are disseminated in hospitals worldwide (Del Barrio-Tofiño et al., 2020). They are characterized by the increasing prevalence of transferable ARGs, particularly those encoding carbapenemases (such as *bla*IMP or *bla*VIM), ESBLs, and defective outer membrane porins.

Antibiotic-resistant *P. aeruginosa* isolates are highly concentrated in hospital effluents (Kerr and Snelling, 2009; Slekovec et al., 2012; Hocquet et al., 2016) and are continuously discharged into natural water basins mainly through sewage and further possibly spread to the soil through natural fertilizers (Topp et al., 2018; Manaia et al., 2022; Marutescu et al., 2022). Slekovec et al. (2012) reported high levels of *P. aeruginosa* in WWTP sludge (2.95 × 106 CFU/kg), within the general range found in hospital wastewater. The treated effluent of three hospital WWTPs was demonstrated to discharge MDR *P. aeruginosa* strains into a city river from Brazil, the major tributary of the Amazon river. The *P. aeruginosa* isolates from the respective river exhibited resistance profiles matching some of the resistant *P. aeruginosa* in the discharged effluent, suggesting that the hospital WWTPs pose a public health risk to residents that live in contact with this stream (Magalhães et al., 2016). The high-risk *P. aeruginosa* clones ST235, ST111, and ST395 were identified throughout the wastewater network of Besançon (France) and recovered from treated water and in the river downstream (Slekovec et al., 2012). Müller et al. (2018) reported MDR and XDR *P. aeruginosa* isolates in the clinical/urban system in Germany belonging to the epidemic outbreak clones ST235, mainly isolated from the undiluted clinical wastewater, with only one strain detected in the effluent of the WWTP, with ST111. These clones can also produce a strong biofilm, likely increasing their capacity to colonize sewer and drinking water plumbing systems (Müller et al., 2018).

Wastewater treatment plants receiving hospital wastewater have also been shown to be an essential source of carbapenem-resistant *P. aeruginosa*. They may therefore contribute to the environmental dissemination of resistance to this important class of antibiotics. In a longitudinal study, carbapenem-resistant bacteria were isolated from the wastewater of a maximum-care hospital for 2 years. The largest ST235 *P. aeruginosa* cluster contained WWTP effluent strains, suggesting the dissemination of this high-risk clone associated with severe infections into the environment (Kehl et al., 2022). Investigation of genetic relatedness between *P. aeruginosa* strains from wastewater treatment (WWT) lagoons and hospital-associated *P. aeruginosa*, as well as community-acquired clones collected in the same geographic area, confirmed the ability of some of these clones to survive in and disseminate from WWT lagoons ponds to connected streams. The WWT lagoons were colonized by highly diverse P. aeruginosa, with most genotypes harboring virulence genes involved in human colonization and infection (Petit et al., 2013). By contrast, Golle et al. (2017) have shown little overlap between a diverse population of carbapenem-resistant P. aeruginosa from WWTP influents and clinical genotypes (Golle et al., 2017). Also, Fuentefria et al. (2011) have found differences in resistance patterns, and frequency of MDR strains among *P. aeruginosa* from the hospital wastewater (Rio Grande do Sul, RS, Brazil) and surface water, the MDR strains being more frequent in the hospital (Fuentefria et al., 2011). In a Polish study, the cultivable *Pseudomonas* strains sampled from raw and treated wastewater and the receiving coastal waters of the Puck Bay, Baltic Sea, exhibited a low prevalence of carbapenems (meropenem and imipenem) resistance and of clinically relevant ESBLs (Luczkiewicz et al., 2015). The analysis of MDR *P. aeruginosa* strains isolated during two consecutive years (2018 and 2019) from hospital settings and hospital collecting sewage tanks. WWTPs located in the central geographical regions of Romania have revealed the presence of epidemic clones ST233, and ST357 in the wastewater and the release of *P. aeruginosa* strains belonging to ST357, ST640c, and ST621 from hospitals into the wastewaters (Gheorghe-Barbu et al., 2022).

Multidrug and carbapenem-resistant *P. aeruginosa* strains have been detected in wastewater in different geographical regions, with contrasting results regarding the relatedness between carbapenem-resistant strains found in wastewater and clinical strains. The variations in the local biodiversity of the aquatic microbial communities, pollution sources, and sampling and analysis methods could explain these discrepancies.

### 2.6. *Enterobacteriaceae*

Different studies report that *Enterobacteriaceae* strains collected downstream of WWTP discharge points can be resistant to different classes of antibiotics, the most frequently reported for *E. coli* isolates being resistance to penicillin (ampicillin, piperacillin, and amoxicillin plus clavulanate) associated with AmpC or ESBLs production, fluoroquinolones (ciprofloxacin and levofloxacin) as well as to trimethoprim/sulfamethoxazole and tetracycline (Amador et al., 2015; Kotlarska et al., 2015; Voigt et al., 2019; Gumede et al., 2021).

The ESBL and quinolone resistance genes found in *E. coli* and *K. pneumoniae* isolates from WWTP raw and treated water are frequently located on class 1 integrons with various gene cassette arrays as well as on IncP-1 and IncFIB plasmids, proving their high risk of spread to human and natural environments (Alouache et al., 2014; Hassen et al., 2021). The NDM-5 carbapenemase producer *E. coli* ST617 was reported in the effluent of a WWTP discharged into the river Rhine (Zurfluh et al., 2017), suggesting that *E. coli* resistant to carbapenems may be present in the community and are released into the aquatic environment with WWTP effluents.

Many studies identify hospital sewage as a more critical source of MDR enterobacterial strains, including carbapenem-resistant isolates, as compared to municipal wastewater (likely due to the selection pressure of antibiotic residues and biocides and higher fecal carriage of ARB in hospitals), underscoring the necessity of an appropriate treatment of the hospitals and other clinical settings wastewater prior to its discharge (Hutinel et al., 2019; King et al., 2020; Addae-Nuku et al., 2022; Mutuku et al., 2022).

Within the past 20 years, a clone of the sequence type ST131 has become the predominant MDR extraintestinal *E. coli* human pathogen (ExPEC) globally due to its ability to acquire antibiotic resistance (Croxen and Finlay, 2010). Thus, ExPEC strains show resistance to fluoroquinolones, extended-spectrum cephalosporins, primarily associated with CTX-M-15 (Coque et al., 2008) and carbapenems (O’Hara et al., 2014; Ortega et al., 2016; Stoesser et al., 2016). In addition, ST131 isolates were identified in an urban WWTP in the Czech Republic (Jamborova et al., 2018), in Norway (Paulshus et al., 2019), in Canada (Finn et al., 2020), in Nigeria (Ben Said et al., 2016), in the surface waters of the rural catchment area in Germany (Müller et al., 2018), hospital wastewater from Japan (Gomi et al., 2017), and in a wastewater treatment network in France (Bréchet et al., 2014).

Due to its capacity to maintain and transmit MGEs, *K. pneumoniae* is positioned as a critical trafficker for amplifying and spreading ARGs among the different environmental niches (Lai et al., 2019), including those encoding for last-resort antibiotics, such as carbapenems or colistin (Galarde-López et al., 2022). Colistin- and/or tigecycline-resistant *K. pneumoniae* ST29 strains, harboring the Tn21-like mercury resistance operon transposons and silver, copper, and arsenic resistance were detected in the WWTP influents from Japan (Hayashi et al., 2021). Lepuschitz et al. (2019) reported that water samples from rivers upstream of Australian cities were negative for ESBL and carbapenemase-producing *K. pneumoniae*. In contrast, all samples taken one to three kilometers downstream of the same cities’ WWTP release points were positive, demonstrating the impact of wastewater effluents and anthropogenic pollution on the aquatic environment (Amos et al., 2014).

Resistance to carbapenems is conferred mainly by the carbapenemase genes *bla*KPC, *bla*NDM, and *bla*OXA, carried by plasmids and transposons. Over 100 different *K. pneumoniae* STs clones have been described to carry *bla*KPC genes (Munoz-Price et al., 2013). The world’s widespread KPC-producing *K. pneumoniae* ST258 emerged in the 2000s as an important human pathogen in urinary and respiratory tract infections. The KPC-2-producing *K. pneumoniae* belonging to the international CC258 has been detected in the treated effluent of an urban WWTP receiving hospital wastewater (Zurfluh et al., 2017). The MDR, KPC-2-producing *K. pneumoniae* ST11, has been isolated from a sampling site in Tokyo Bay, Japan, near a WWTP (Sekizuka et al., 2018). A study in Puerto Rico 6 months after Hurricane Maria, a category V storm, revealed various clinically significant mobile β-lactam ARGs downstream of WWTP discharge, including KPC-2 within an ISKpn6-like transposase (Davis et al., 2020).

An NDM-1-producing *K. pneumoniae* ST147 clone was reported in the wastewater of a WWTP in Basel (Switzerland), most probably originating in the wastewater from clinical settings (Nüesch-Inderbinen et al., 2018). In addition, Surleac et al. (2020) described carbapenemases-producing *K. pneumoniae* ST35, ST219, ST364, ST395, ST485, and ST1878 in wastewaters collected from WWTPs in Southern Romania, of which ST395 has clinical importance, while ST35 and ST485 are sporadically related to clinical cases.

Hypervirulent *K. pneumoniae* strains are recognized as an urgent threat to human health, producing community-associated invasive diseases that can affect young, healthy people and hospital-acquired infections. In clinical settings, hypervirulent *K. pneumoniae* has a marked propensity to acquire antibiotic resistance and rarely carries virulence plasmids (Turton et al., 2019). The ST147 *K. pneumoniae* high-risk clones (*bla*NDM/*bla*OXA-48) have been identified in the wastewater of a full-care hospital, and the effluent of the WWTP receiving this wastewater, suggesting the persistence of this clone during the wastewater treatment in a study performed in Germany (Kehl et al., 2022). Also, the transmission of an MDR *K. pneumoniae* ST101 clone from hospital to wastewater and its persistence after chlorine treatment was demonstrated in Romania. The strains belonging to this clone harbored multiple acquired ARGs (incl. *bla*CTX-M- 15, *bla*OXA- 48) and chromosomal mutations involved in antibiotic resistance. Twenty-nine virulence genes were identified in iron acquisition, biofilm, pili formation, adherence, and the type six secretion system–T6SS-III (Popa et al., 2021). The persistence of carbapenemase-producing *K. pneumoniae* from hospital to environment via municipal WWTP has also been found in a study performed in the US (Loudermilk et al., 2022). A recent study has demonstrated that carbapenemase-producing, hyper-virulent *K. pneumoniae* strains could be transmitted from wastewater via bioaerosols to the upper respiratory tract of WWTP employees (Rolbiecki et al., 2021). All these studies demonstrate that the WWTPs contribute to the transmission and spreading of MDR *Enterobacteriaceae* in the aquatic environment, posing a risk for the communities living in proximity and, eventually, for the WWTP workers.

## 3. WWTPs effluents–A source of ESCAPE-related ARGs into the aquatic environment

ESCAPE-related ARGs, encoding every known type of mechanism (target protection, target modification, drug modification, reduced permeability or efflux), have been widely reported in both wastewater and downstream environments across continents (Freeman et al., 2018; Pazda et al., 2019; Surleac et al., 2020; Popa et al., 2021; Gheorghe-Barbu et al., 2022). These ARGs have been detected in bacterial isolates or total genomic DNA samples (Table 1).

**TABLE 1:** ESCAPE-related ARGs into the aquatic environment.

Species	Location	Isolation source	ARGs	MGEs	References
Not provided	Wascana Lake, Canada	Surface water samples	*sul1*, *ermB*, *blaCTX-M*, *tetO*, *ermB*, *sul1*, *qnrS*	*intI*, *intI1*	Freeman et al., 2018
*K. pneumoniae*	Romania	WWTP influent and effluent	*bla*_SHV_, *bla*_OXA_, *bla*_TEM_ and *bla*_CTX_, *bla*_NDM–1_, *bla*_OXA–48_, *bla*_KPC–2_, *aac(6′)*, *ant(2″)Ia*, *aph(3′)*, *aaD*, *aac(3)*, *aph(6)*	*qacE*Δ*1* integron associated gene	Surleac et al., 2020
*A. baumannii* and *P. aeruginosa*	Romania	Hospital settings and collecting sewage tanks, WWTPs	*bla*_OXA23_, *bla*_OXA24_, *bla*_SHV_, *bla*_TEM_, *bla*_GES_, *bla*_IMP_, *bla*_VIM_, *bla*_NDM_, *bla*_VEB_	*qacE*Δ*1* integron associated gene	Gheorghe-Barbu et al., 2022
Not provided	Germany	Influent and effluent of 62 WWTPs	*sul1*, *ermB*, *tetM*, *sul2*, *qnrS*, *bla*_*CTX–M*_	*intI1*, *korB*	Pallares-Vega et al., 2019
*Enterococcus spp.*	Dresden, Germany	WWTPs	*bla*_*CTX–M–32*_, *bla*_*OXA–58*_, *bla*_*SHV–34*_, *dfrA1*, *sul1*, *sul2*, *tetM*, *vanA*	–	Caucci et al., 2016
*E. coli*, *K. pneumoniae*, *P. aeruginosa*, *Acinetobacter* spp., *S. pneumoniae*, *S. aureus*, and enterococci	30 European countries	Urban WWTPs	*aadA*, *strB*, *bla*_*GES*_, *bla*_*OXA*_, *bla*_*VEB*_, *bla*_*NDM*–_*_1_*, *bla*_*KPC*_, *bla*_*VIM*_, *bla*_*IMP*_, *mcr-1*, *mecA*, *vanA*, *ereA*, *ermF*, *matA*, *sul1 tetM* and *tetQ*	*qacEdelta1*, *qacH*, *intI1*, *tnpA*, *Tp614*, *ISAba3*, *ISPps*, and *ISSm2*	Pärnänen et al., 2019
*E. coli*, *K. pneumoniae*	Germany	WWTPs	*mcr-1*, *ermB*, bla*_*CTX–M*_*_–32_, *bla*_*TEM*_, *bla*_*CMY*–2_, bla*_*CTX–M*_*, *tetM*	–	Hembach et al., 2017
Not provided	10 European countries	16 Urban WWTPs	*bla*_TEM_, *bla*_OXA–48_, *bla*_OXA–58_, *bla*_CTX–M–15,_ *bla*_CTX–M–32,_ *bla*_KPC–3_, *sul1*, *tetM*, *mcr*-1	*intI1*	Cacace et al., 2019
Not provided	Danube River Basin, 9 European countries	WWTPs	*aph*(III)a, *bla*_OXA_, *bla*_SHV_, *erm*B, *erm*F, *mec*A, *qnr*S, *sul*1, *tet*B, *tet*M, and *van*B	*intI1*	Alygizakis et al., 2019
*S. aureus*	Olsztyn, Poland	WWTP	*mecA*, *vanA*, *nuc*	*qac*A/B	Zieliński et al., 2020
*Acinetobacter spp.*, *Pseudomonas spp.*, *Clostridium spp.*	Spain	WWTP	*sul*1, *sul*2, *erm*B, *tet*W, *tet*M, *qnrS*, *bla*_*TEM*_, *bla*_KPC_	*intI1*	Auguet et al., 2017
*A. baumannii*	Tennessee, United States	WWTP	*msrE*, *mphE*, *tet*(39), *cfxA6*, *oxa280*, and *aadA4*	–	Murphy et al., 2021
*A. baumannii*, *P. aeruginosa*, *E. cloacae*	Republic of Korea	12 WWTPs	*bacA*, *aph(3″)-Ib*, *sul1 sul1*, *aac(6′)-31*, *aadA5*, *aadA22*, *rbpA*, *qnrB40*, *mdtG*, *aac(6′)*, *tet(39)*, *emrA*, *mphG*, *aacA4*, *cpxA*, *mupA*, and *macA*	*qacH*	Raza et al., 2022

Dutch WWTPs were shown to discharge on average 106 copies of ARGs per liter of effluent to the receiving water bodies, the dominant ones being the ARGs to sulphonamides (sul1) and macrolides (*erm*B), their high occurrence being possibly associated with MGEs or might be due to the prolonged use of these antibiotics in humans and animals (Pallares-Vega et al., 2019). Some ARGs, like the resistance to sulfonamides (sul1, sul2), glycopeptides (*van*A), and tetracycline (tetM), were shown to be more abundant in treated wastewater, a fact that might also be explained by a long history of usage of sulphonamide and tetracycline antibiotics (Heuer et al., 2011; Caucci et al., 2016; Pärnänen et al., 2019). Several other ARGs conferring resistance to different classes of antibiotics, including macrolide-lincosamide-streptogramin B (MLSB) (*erm*A, *erm*B), beta-lactams (*bla*SHV-02, *bla*VEB, *bla*OXA-10, *bla*CTX-M-32, *bla*TEM, and *bla*CMY-2), glycopeptides (vanA), aminoglycosides (aadA, aadA1, and aadA2) and also multidrug resistance (*qac*Edelta1) were significantly enriched in effluent wastewater from Chinese and European WWTPs (Hembach et al., 2017). It has been hypothesized that a positive selection or community change during wastewater treatment could enrich *van*A (Pärnänen et al., 2019). Worrisome is the detection of the colistin resistance gene *mcr*-1 in influent samples of seven WWTPs investigated in Germany and its release into the aquatic environment (Hembach et al., 2017).

The absolute abundance of nine ARGs and a class 1 integron-associated integrase gene in the wastewater effluents of 16 WWTPs from ten different European countries could be ranked along the following order: *int1* > *sul1* > *tet*M > *bla*_OXA–58_ > *bla*_TEM_ > *bla*_OXA–48_ > *bla*_CTX–M–32_ > *mcr-*1 > *bla*_CTX–M–15_ > *bla*_KPC–3_ (Cacace et al., 2019). Downstream of the plants, these ARGs were quantifiable in many cases, suggesting an impact of the effluent on the receiving waterbody (Cacace et al., 2019). The *aph(III)a*, *bla*_OXA_, *erm*B, *erm*F, *sul1*, and *tet*M resistance genes had a wide-spread occurrence in effluents discharged from 12 WWTPs in the Danube River Basin (Romania, Serbia, Hungary, Slovenia, Croatia, Slovakia, Czechia, Austria, Germany); in this study, the highest absolute concentrations (gene copies/mL) were observed for four ARGs (*aph(III)a*, *tet*M, *van*A, and *mec*A) at the WWTP Bucharest (Romania), four ARGs (*bla*_OXA_, *bla*_SHV_, *qnr*S, and *tet*B) at the WWTP Sabac (Serbia), two ARGs (*erm*B, *sul*1) at the WWTP Varazdin (Croatia) and one ARG (*erm*F) at the WWTP Brno (Czechia) (Alygizakis et al., 2019).

The analysis of the abundance of resistance and virulence genes in *Staphylococcus* spp. strains from untreated and treated wastewater, an activated sludge (AS) bioreactor, and from surface water collected upstream and downstream the WWTP, revealed that *S. aureus* was present in 63% of the samples, with 20% of the strains carrying the *van*A gene. The *hla* virulence gene was present in most of the isolates (80%), followed by the PVL gene (Zieliński et al., 2020). The highest abundance of virulence and resistance genes among *S. aureus* strains was observed in the untreated wastewater, gradually decreasing in treated water and downstream river water, probably due to the synergic effect of wastewater treatment, dilution, as well as influence location-specific abiotic and biotic factors, such as temperature, pH, solar radiation, mineral and organic content, microbial community structure, seasonal influence, competition, temporal variations in the prescription level of antibiotics, etc. (Caucci et al., 2016; Auguet et al., 2017; Zieliński et al., 2020).

The analysis of the water metagenomes sampled immediately downstream, and at 6.4 km from a WWTP effluent released into the surface water of a small river located in the rural area of Knox County, Ohio, showed that the metagenome just downstream of the WWTP effluent was substantially enriched in 15 different ARGs relative to the remote location. Among these enriched ARGs were 6 ARGs commonly associated with *A. baumannii*, such as *msr*E, *mph*E (macrolide resistance), and *tet* (tetracycline resistance), that persisted 6.4 km downriver. In addition, the samples from directly downstream of the WWTP were enriched in *Acinetobacter* spp. and gut-associated taxa: *Bacteroides* and *Firmicutes*. The ARG levels, taxa composition, and prevalence were independent of the seasonal effluent chlorination and nitrogen and phosphorus concentrations (Murphy et al., 2021).

Using a hiseq-based metagenomic sequencing approach to investigate the core resistome of urban WWTPs persisting through wastewater treatment processes, the influents and effluents of 12 urban WWTPs in Republic of Korea were analyzed. The abundance of some core ARGs such as macrolide-lincosamide-streptogramin–and tetracycline-resistance genes was higher in the influent samples, while others such as sul1, APH(3″)-lb, and RbpA were more abundant in the effluent. Most core ARGs were carried by ESCAPE pathogens, such as *A. baumannii*, *E. cloacae*, and *P. aeruginosa*. Also, the abundance of core ARGs in the effluent was correlated with an abundance of phages, suggesting a role of transduction in disseminating ARGs from WWTP effluent to surface water (Raza et al., 2022).

Generally, conventional WWTP treatments can reduce ARGs’ overall richness, concentration, and relative abundance in variable proportions (Yang et al., 2014; Pallares-Vega et al., 2019; Li et al., 2022). The primary treatment is usually not efficient in removing pathogens and ARB, requiring the use of advanced treatments (e.g., sand and membrane filtration) or disinfection methodologies (e.g., chlorination, UV radiation, and ozonation) after conventional activated sludge treatment (Hazra and Durso, 2022). Many WWTPs only use conventional activated sludge for wastewater treatment, which, however, has been shown to have better removed fecal coliforms, enterococci, and *E. coli* in comparison to septic tank systems; but, despite this reduction, an increase in the MDR profiles has been observed during wastewater treatment (Barrios-Hernández et al., 2019; Hazra and Durso, 2022). From an analysis of the influent concentrations and the removal of ARGs in a large number of WWTPs in the Netherlands, the researchers concluded that WWTP design parameters such as size, presence of primary clarification, type of phosphorus removal, and operational parameters such as hydraulic retention time, sludge retention time, anaerobic contact time and effluent total suspended solids did not affect the removal of the studied ARGs and MGEs significantly (Pallares-Vega et al., 2019). Disinfection steps, i.e., chlorination (Mao et al., 2015; Yuan et al., 2015), UV irradiation (Sousa et al., 2017), and ozone (Alexander et al., 2016), alone or in combination (UV and chlorination) (Zhang et al., 2015), were applied in WWTPs to assess their efficiency in removing ARB and ARGs. The results showed that they were not able to altogether remove the ARGs. Moreover, some of the cleaning methods could increase the proportion of ARB through the alteration of bacterial cell permeability by the activation of efflux pumps, accumulation of intermediate disinfection by-products, and promotion of HGT, particularly in case of prolonged contact with low chlorine concentrations (Huang et al., 2011). Chlorination could result in the survival of the ARB in more significant percentages due to their different susceptibility to disinfection (Mao et al., 2015). The ozone treatment causes DNA damage but has a reduced effect on guanine-rich DNA (Alexander et al., 2016). UV light is partially absorbed by organic and inorganic matter present in wastewater; thus, the DNA damage depends on the UV dosage; additionally, different sensitivity of ARB to UV radiation was noticed (Hazra and Durso, 2022). A membrane bioreactor has been shown to increase the municipal WWTP efficiency for decreasing the abundance of ARGs and loads of *E. coli*, enterococci, and *P. aeruginosa* strains (Ng et al., 2019). Still, the ARGs of 12 antibiotic classes, including aminoglycosides, beta-lactams, macrolides-lincosamides-streptogramins, polymyxins, quinolones, rifamycin, tetracyclines, and multidrug resistance, persisted during treatment. Constructed wetlands (CWs) seem to be a sustainable “green” wastewater treatment option that can be used as an alternative to WWTP or as a component of conventional WWTP, having the ability to remove antibiotics, ARB, and ARGs (Dires et al., 2018; de Oliveira et al., 2019; Riaz et al., 2020; Hazra and Durso, 2022). CWs can be used alone or even after primary treatment, contributing to 2–4 log pathogens reduction (Sleytr et al., 2007; Karimi et al., 2014) and increasing the performance of WWTP when coupled as secondary/tertiary units to primary treatment (Chen et al., 2014).

A recent comprehensive review on the performance of primary, secondary and tertiary treatments of wastewater in removing antibiotics, bacterial pathogens, ARB and ARGs concludes that combinations of different methods are more efficient in the elimination of a large number of ARB (Hazra and Durso, 2022).

## 4. Conclusion and perspectives

In this review, we have focused on the relationships between ESCAPE pathogens and the WWTPs. Different studies performed in different geographical areas report that ESCAPE pathogens and their associated ARGs, including high-risk clones and resistance determinants to last resort antibiotics such as carbapenems and colistin, as well as MDR platforms are present in wastewater. The sources for the enrichment of ESCAPE pathogens are diverse, including clinical settings, animal husbandry facilities, urban and municipal wastewater systems, etc. Some studies are demonstrating the clonal relationships and dissemination of ESCAPE clinical strains, such as *A. baumannii*, *P. aeruginosa* and *K. pneumoniae* into the wastewater via hospital effluents. Also, WWTPs seem to provide a suitable environment for the enrichment of resistance and virulence determinants in ESCAPE bacteria, such as enterococci and *S. aureus*. Therefore, the efficiency of different wastewater treatment processes and combinations thereof regarding the removal of clinically relevant ARB species and ARGs, as well as the influence of water quality factors on their performance should be explored and monitored. Also, the development of more effective disinfection and treatment methods could contribute to prevent or reduce the release of antibiotics, ARB and ARGs into the natural environment via WWTP effluents. In order to achieve these goals, research is needed to select the most appropriate indicators (ESCAPE bacteria and/or ARGs) and standard methods for assessing them, to facilitate the comparison of resistance levels between countries and different WWTPs (Uluseker et al., 2021). The quantification of known ARGs along the wastewater treatment chain need to be complemented with information regarding the origin of these genes, their genetic context, the type and level of selective pressure, potential for HGT and compatibility with putative pathogenic hosts (Bengtsson-Palme and Larsson, 2015). Also, the possible use of current microbiological indicator species such as enterococci and *E. coli* in managing the risks associated with the environmental resistome, depending on the total counts as well as the resistance level in these strains, needs to be explored in the future. This knowledge will allow to establish quality threshold limits regarding the AR for point sources and effluents that are being directly discharged to water bodies and agricultural land for irrigation, and ultimately to reinforce the barrier role of WWTPs against environmental and public health threats.

## Author contributions

LGM and MCC conceived the review. LGM, MP, IG-B, ICB, GPG, and COV wrote the draft and drown the figure. DR-M, FB, HB, C-FF, MAK, BS, LW, DGJL, DN, KR, AMRH, AW, and HS completed and corrected the drafts. MCC integrated all the comments from the authors and assembled the final form of the review. All authors contributed to the article and approved the submitted version.

## Footnotes

1
https://www.jpiamr.eu
